# Do EEG and Startle Reflex Modulation Vary with Self-Reported Aggression in Response to Violent Images?

**DOI:** 10.3390/brainsci9110298

**Published:** 2019-10-30

**Authors:** Sajeev Kunaharan, Sean Halpin, Thiagarajan Sitharthan, Peter Walla

**Affiliations:** 1 School of Psychology, Centre for Translational Neuroscience and Mental Health Research, University of Newcastle, Callaghan, NSW 2308, Australia; Sajeev.kunaharan@uon.edu.au (S.K.); Sean.halpin@newcastle.edu.au (S.H.); 2 Sydney Medical School, University of Sydney, Sydney, Camperdown, NSW 2050, Australia; thiagarajan.sitharthan@sydney.edu.au; 3 CanBeLab, Department of Psychology, Webster Vienna Private University, Palais Wenkheim, 1020 Vienna, Austria

**Keywords:** event-related potentials (ERPs), P300, aggression, startle reflex modulation (SRM), self-report, emotion

## Abstract

Increased violence and aggressive tendencies are a problem in much of the world and are often symptomatic of many other neurological and psychiatric conditions. Among clinicians, current methods of diagnosis of problem aggressive behaviour rely heavily on the use of self-report measures as described by the Diagnostic and Statistical Manual of Mental Disorders 5th Edition (DSM-5) and International Classification of Diseases 10th revision (ICD-10). This approach does not place adequate emphasis on objective measures that are potentially sensitive to processes not feeding into subjective self-report. Numerous studies provide evidence that attitudes and affective content can be processed without leading to verbalised output. This exploratory study aimed to determine whether individuals in the normal population, grouped by self-reported aggression, differed in subjective versus objective affective processing. Participants (*N* = 52) were grouped based on their responses to the Buss–Durkee Hostility Inventory. They were then presented with affect-inducing images while brain event-related potentials (ERPs) and startle reflex modulation (SRM) were recorded to determine non-language-based processes. Explicit valence and arousal ratings for each image were taken to determine subjective affective effects. Results indicated no significant group differences for explicit ratings and SRM. However, ERP results demonstrated significant group differences between the ‘pleasant’ and ‘violent’ emotion condition in the frontal, central and parietal areas across both hemispheres. These findings suggest that parts of the brain process affective stimuli different to what conscious appraisal comes up with in participants varying in self-reported aggression.

## 1. Introduction

Violent behaviour is a major social and public health problem in most parts of the world [1]. Consequently, the identification and effective management of individuals who present with violent or aggressive behaviours are of paramount importance. Aggression can be symptomatic of a variety of underlying psychiatric and neurological illnesses [1,2]. Aggression is also shaped by culture, the environment and societal factors [3]. To date, many clinicians and researchers have aimed to assess the effects and extent of aggression in individuals using self-report measures to determine whether individuals meet diagnostic criteria as described in the Diagnostic and Statistical Manual of Mental Disorders 5th Edition (DSM-5) [4] and the International Classification of Diseases 10th revision (ICD-10) [5]. These methods of assessment comprise almost exclusively of traditional questionnaires and face-to-face interviews, allowing the clinician or researcher to make a diagnosis. A major weakness of these methods is that, even when several measures are taken, they rely predominantly on conscious self-report, which has been shown to have inherent biases mainly due its subjective nature [6]. These biases are most highly evident when the individual discusses behaviour subject to social disapproval, termed social desirability bias [7,8,9]. Conscious explicit responses (derived from questionnaires and face-to-face interviews) can be modified over several stages of processing and may not accurately reflect what might be occurring on a level below conscious awareness. Several papers have pinpointed the influence the non-conscious has on our consciously perceived reality [10,11,12,13,14]. One solution may be to try to identify neural substrates and mechanisms underlying the expression of aggressive behaviour [1].

It is important to recognise that aggression is by no means a unimodal behaviour. Several authors have postulated various types of aggression. For example, Moyer [15] examined the following aggression sub-categories: fear-induced aggression, maternal aggression, inter-male aggression, sexual aggression, irritable aggression, predatory aggression and territorial aggression. Many of these terms were formulated during animal studies in aggression; Siegel and Victoroff [1] state that human aggression is even harder to categorise as any one of the abovementioned categories could involve one or more types of aggressive behaviour. Siegel and Victoroff argued that the varieties of aggression listed above may be reduced to two main categories: affective aggression (which may also be called reactive, defensive or hostile aggression) and predatory aggression (which may be referred to as impulsive, proactive, premeditated or instrumental aggression). Predatory aggression differs from affective aggression, which is mostly associated with a threat or fear. In the present study, however, we determined self-report aggression as a unitary construct and did not categorise groups based on impulsivity. However, the Barratt impulsiveness scale [16] was provided to participants to determine if the cohort was more likely to be impulsive in nature if they self-reported higher aggressive tendencies.

As aggressive behaviour is often symptomatic of many other clinical disorders, clinicians may be faced with the challenge of assessing the presence and extent of aggression and aggressive subtypes. The Diagnostic and Statistical Manual of Mental Disorders 5th Edition [4] (DSM-5) and the International Classification of Diseases 10th revision (ICD-10) [5] list behavioural and cognitive symptoms, the presence of which are typically assessed using self-report, behavioural observation and clinical interviewing. Although such methods are a useful way to determine an individual’s conscious (subjective) emotional state, these methodologies are subject to biases through conscious deception or even the fact that emotional cognitive processes and biases can occur without conscious awareness [13,14,17]. The act of explicitly delivering responses requires conscious cognitive processing, which incorporates a coordinated use of sub-cortical brain processes and conscious cortical brain processing. Walla et al. [11] labelled this phenomenon cognitive pollution.

It has been demonstrated that information consciously reported via self-report does not always correspond to what is recorded objectively. For the purpose of this study objectively means recorded not through verbal report, but via some sort of objective physiological measure. It is also assumed that physiological measures (e.g., electroencephalography (EEG), startle reflex modulation (SRM), etc.) that are sensitive to neural processing have the potential to reflect information processing outside language access, which in turn can be labelled non-conscious. However, of course we cannot claim that anything recorded objectively is naturally non-conscious. Regarding discrepancies between self-report (subjective) and objective measures a lot of insight so far has come from studies that utilised startle reflex modulation (SRM) to present such findings. SRM is a technique in which a short, unexpected burst of auditory white noise (usually around 100–110 dB) is presented to each ear of unsuspecting participants while they are exposed to a controlled foreground stimulus depicting varying affective content [18]. The resultant startle response, measured by the intensity and latency of the eye blink reflex, pertains to raw affective information processing and is usually determined by the appetitive nature of the affective foreground stimulus. Lang et al. [19] showed that the magnitude of the eye blink recorded resulted in smaller blinks if the foreground stimulus was appetitive and larger when the foreground stimulus was aversive.

In studies using SRM, raw affective state has been investigated in various different contexts such as marketing, walking in urban environments, schizophrenia, psychopathy, food preferences and disabilities [20]. SRM has also been suggested as an additional technique for various disciplines outside the neurosciences [21,22,23]. Grahl et al. [24] utilised both self-report measures and SRM to determine if bottle shape elicits a variation in gender-specific affective responses. They showed that although subjective self-reports showed no differences between males and females, a significant gender effect was noted in the SRM blink amplitude of male respondents to a particular shape of bottle. They concluded that physiological measures of raw affective responses may be sensitive to emotional cues which are not revealed by conscious self-report. Additionally, it was shown that participants altered their behaviour depending on whether they were subliminally exposed to a happy, neutral or angry face stimulus [12]. By these authors it was posited that the subliminally presented emotion faces caused affective reactions which altered behaviour without conscious awareness. Further studies involve those done by Allen, Trinder and Brennan [25]. They investigated the modulation of the startle effect in depressed and non-depressed participants and showed that the self-reported valence of presented images remained similar across both depressed and non-depressed groups. However, the SRM demonstrated clear between group differences. Another recent study by Walla, Koller, Brenner and Bosshard [26] used varying rounds of evaluative conditioning of liked and disliked brands and found that conditioning effects resulted in selective differences in self-report versus SRM measures. These findings further support the notion that objectively accessible processes play a pivotal role in our behaviour regardless of conscious awareness.

It appears that questionnaires and other self-report tools can provide quick and reliable information about cognitive processes occurring on a conscious level. However, they only capture what may be a fraction of an individual’s psyche. It is therefore timely to examine whether we can use objective physiological measures to observe differences in brain processes that are inaccessible when using conscious self-report measures.

Event-related potentials (ERPs) provide a method by which we can examine language-independent processes that are not necessarily leading to actual behaviour and yet reflect information processing involved in decision-making processes. ERPs provide an easily obtainable and non-invasive method to study neural activity and, due to ERPs advantages in temporal resolution, real-time discrimination of cortical processes [27]. Studies examining the ERPs of aggressive individuals predominantly identify the P300 component as a marker of aggression. The P300 component appears to reflect perceptual processing used to identify stimulus relevance [28]. This includes latency, which measures evaluation time, and amplitude, which relates to the ability to process a stimulus. Lower amplitude P300 is suggestive of less efficient cognitive functioning [29]. The P300 wave is associated with higher order cognitive functioning, and working memory is typically reduced in those who present with impulsive aggressive tendencies when presented with an oddball task [27,30,31]. Gerstle et al. [32] demonstrated that within the normal population, individuals who self-reported as being impulsive aggressive presented with significantly lower P300 amplitude in frontal electrode sites compared with nonaggressive controls. In contrast, individuals who presented as being premeditated aggressors did not show the reduced P300 component characteristic of impulsively aggressive individuals [33,34]. This suggests that the P300 could serve as a marker specific to individuals who present with aggression of an impulsive nature [35].

The N400 component of the ERP has also been studied in populations of aggressive individuals. Gagnon et al. [36] measured the neural activity associated with violating expectations about hostile and non-hostile intentions in aggressive and non-aggressive groups. Aggressive individuals produced a larger N400 response compared to controls when exposed to a critical word which violated a hostile expectation. Additionally, aggressive individuals showed an enhanced late positive potential (LPP) when hostile scenarios took place in a non-hostile context. The LPP is a sustained positivity in the ERP usually in the central and parietal regions of the scalp, and it is a marker for processing affective content [37]. In general, a larger LPP effect for individuals who report as being more aggressive has been observed [36]. Most studies tend to focus on the P300 component as it is considered to be a more reliable indicator of violence [31], which is why we will focus on this ERP component in the discussion section (see below).

A search of the literature revealed no studies investigating startle effects of violent or aggressive individuals within non-clinical populations. In all cases where startle was studied, the target behaviour (aggression/violence) was symptomatic of some other disorder. Several studies have utilised SRM to study particular clinical groups which present with violence and/or aggression as a symptom of their condition, these being criminal psychopathy [38,39], schizophrenia [40,41], antisocial personality disorder [39,41,42] and borderline personality disorder [43], among others. Hazlett et al. [44] showed that patients with borderline personality disorder (BPD) exhibited larger eye blink responses to unpleasant but not neutral words. The authors suggested that individuals suffering from BPD respond to aversive stimuli as a result of an exaggerated physiological effect. Patrick et al. [38] used SRM to determine the emotional responding of criminal psychopaths and found the psychopathy group did not show the clear linear relationship between slide valences and startle magnitude as was evident in the non-psychopathic and mixed subject controls. This suggested the psychopathy group had an abnormality in the processing of emotional stimuli.

The present study aimed to determine whether individuals present with differences between their subjective self-reported measures and objective physiological recordings. It was hypothesised that participants with higher levels of self-reported aggression would show attenuated P300 and LPP amplitudes as well as startle responses to emotion images exhibiting negative affect (‘violent’ and ‘unpleasant’) than participants with lower levels of self-reported aggression. It was also hypothesised that there would be no differences in deliberate self-reported valence and arousal responses to the emotion images between participants with high aggression and lower aggression, which is based on previous findings that showed physiological effects in the absence of behavioural effects regarding emotion-related information processing (e.g., [24]). Valence refers to the concept of approach versus withdraw or in other words it reflects positive versus negative aspects of a stimulus whereas arousal means a state along the line between calm and excited.

## 2. Materials and Methods

### 2.1. Participants

Participants were 52 male students enrolled at the University of Newcastle, Australia with an age range between 18 and 30 years (mean (M) = 21.13; standard deviation (SD) = 2.93). Participants reporting an age outside of this range were automatically taken to the end of the survey and were unable to proceed. Participants provided informed written consent and were native English speakers, right-handed, had no known history of neuropathology, no history of being a victim of physical/sexual abuse, no history of being incarcerated in a penitentiary and were not taking any nervous system targeting medication such as stimulants or anti-depressants. The project was approved by the University of Newcastle Human Research Ethics Committee (H-2013-0309).

### 2.2. Participant Demographics

Our cohort consisted of a largely homogeneous sample of undergraduate and postgraduate students at an Australian University. A majority of the participants were students who had completed at least a secondary school level of education, were either living with a partner or had never been married and identified themselves as being Caucasian born in Australia (see Table 1).

### 2.3. Measures

#### 2.3.1. Online Questionnaire

An online questionnaire was administered to determine self-report measures prior to physiological testing. The online questionnaire was created using Lime Survey [45] and included demographic questions, a modified version of the Buss–Durkee Hostility Inventory (BDHI) [46], Snyder Self-Monitoring Scale-Revised [47] and Barratt Impulsivity Scale (BIS-11) [16]. The BDHI was modified from its original publication by grouping all questions into sub-groups under relevant headings. This was done intentionally to determine if any conscious self-monitoring effects could be seen, as participants would know exactly what component of their own aggressive traits were being measured. This could then be compared to scores obtained by the Snyder Self-Monitoring Scale. Total scores for the modified Buss–Durkee Hostility Inventory were determined by following the scoring protocol outlined by Buss and Durkee [46]. A median split of total BDHI score was utilised to determine ‘high’ and ‘low’ aggressive individuals. The BIS-11 and Snyder self-monitoring questionnaires were used to determine if there was any relationship between total scores for these measures and ‘high’ and ‘low’ aggression. The questionnaire took 20–25 min to complete.

#### 2.3.2. Stimuli

The experiment used 150 images obtained from the International Affective Picture System (IAPS) [48]. Each image was categorised into 1 of 5 emotion categories: violent, neutral, pleasant, unpleasant and erotic. Each emotion category contained 30 images. Each emotion category differed in normative valence (M = 7.3 erotic, 3.22 violent, 5.09 neutral, 7.38 pleasant, 2.01 unpleasant) and arousal (M = 6.76 erotic, 5.94 violent, 4.53 neutral, 5.94 pleasant, 5.95 unpleasant) ratings (scale range 1–9; all normative values based on data acquired from Lang, Bradley and Cuthbert [48]). In order to increase the chance for possible effects, we selected the emotion categories on the basis of differences in valence and arousal combinations. The erotic and pleasant categories have similar valence, but different arousal levels whereas the violent and unpleasant categories have similar arousal, but different valence levels (as taken from the IAPS). This mix in valence/arousal combinations was meant to optimise chances to find possible effects related to self-reported aggression.

#### 2.3.3. EEG Recordings

Brain potential changes were recorded using a 64-channel BioSemiActiveTwo system and ActiView software (BioSemi, Amsterdam, The Netherlands) at a rate of 2048 samples/s. Electrode layout conformed to the international 10–20 standard of electrode placement and all electrodes were finally referenced to a common average signal. Additional electrodes were placed latera-locularly, supra-ocularly, infra-ocularly and on the mastoids for reference and to monitor eye blinks. Data sets were batch processed using EEG-Display (version 6.4.8; Fulham, Newcastle, Australia). During processing the sampling rate was downsampled to 256 samples/s and filtered from 0.1 Hz to 30 Hz. ERP epochs were defined in relation to the presentation of each IAPS image from −100 ms pre-stimulus to 1000 ms post-stimulus onset. All epochs were baseline corrected with the correction occurring 100 ms prior to stimulus onset and data points along the ERP were reduced to 15 data points along the first second post-stimulus presentation.

#### 2.3.4. Startle Reflex Modulation Recordings

Orbicularis oculi muscle potential changes as responses to startle probe presentations were measured. Startle responses were measured using a Nexus-10 (produced by Mind Media BV, Louis Eijssenweg 2B, 6049CD Herten, The Netherlands) recording device and Bio-trace + software. Two 4 mm bipolar electromyography (EMG) electrodes were attached to the left eye of each participant and potential changes of the musculus orbicularis oculi were measured at a sampling rate of 2048/s with a band pass filter of 20–500 Hz during the recording. Electrodes were placed with approximately 20 mm spacing on the inferior orbicularis oculi of the left eye. The raw EMG data was then converted into amplitudes using the root mean square (RMS) calculation. Startle blink amplitude value was defined as the peak rise in the EMG wave across startle trials upon stimulus presentation. Each image category (violent, neutral, pleasant, unpleasant and erotic) contained 5 images which were paired with a startle probe (total 25 startle probes during the experiment). Startle probes were presented binaurally at 110 dB using headphones (Sennheiser HD280; Sennheiser Australia, Chatswood 2067 NSW, Australia).

#### 2.3.5. Explicit Responses

Explicit responses (ratings) during the experimental stage for both valence and arousal were determined by each participant for each image presented. Participants rated each image using a Likert scale from 1–9 for valence (1 = very pleasant; 9 = very unpleasant) and arousal (1 = very intense; 9 = very calming). Scores for each emotion category were averaged for each individual for analytical statistics and then grand averaged across respective ‘high’ and ‘low’ groups for displaying findings.

### 2.4. Experimental Task and Procedure

Upon completing the online questionnaire, participants were asked to complete the remainder of the experiment at the Functional Neuroimaging Laboratory (University of Newcastle, Newcastle, Australia). Each participant was seated approximately 50 cm in front of a 32” light-emitting diode (LED) monitor (resolution 1024 × 768 pixels) and fitted with the Biosemi EEG electrode system and Biotrace electrodes for startle reflex modulation. To visually present instructions and visual stimuli, a computer program presentation (NeuroBehavioral Systems, Albany, NY, USA) was used. Stimuli presentation and all physiological recordings were conducted by the researcher from a separate room.

Each participant was provided with a brief overview of the experiment and asked to read the instructions on the screen before beginning. Headphones were placed over the participant’s ears and the lights in the room were dimmed slightly to allow for adequate focus on the screen. Upon commencement, participants each viewed all 150 IAPS images, randomly ordered, and rated each image based on valence and arousal. Each image was presented for 5 s, followed by the valence rating scale and then the arousal rating scale. Participants were allowed to take their time with the rating of each but were instructed to try not to think about it too much and to provide their most instinctive response. Upon the rating of each scale, a fixation cross appeared in the centre of the screen for 1 s before the following image was presented. Startle probes occurred randomly but with at least a 40 s time window between consecutive startle probes. If and when a startle probe was to be paired with an image it would occur on the 4th second following image onset to ensure adequate affect. Physiological and explicit measures were taken from each participant for all 150 IAPS images with a short break offered to the participants during the halfway point to help reduce the effects of fatigue

### 2.5. Statistical Analysis

The principle analysis for all separate measures (startle responses, valence ratings, arousal ratings, ERPs) was a general linear repeated measures ANOVA (analysis of variance) with a 5 by 2 design with the within-subject factor of emotion including five levels (pleasant, unpleasant, erotic, violent and neutral) and the between-subject factor of each group including two levels (low self-reported aggression, high self-reported aggression). For each event-related potential, of particular interest was the change in ERP amplitude (µV) across frontal, central and parietal regions of the brain calculated for each of the 15 time points separately (in ms after stimulus onset). For ERP analysis, an additional within subject factor of hemisphere (AF3/4, C3/4 and P3/4) was also used to determine hemispheric asymmetry effects. Electrode locations were selected on the basis of prior findings relevant to this study (see [49]). Mean values per emotion category were utilised for the general repeated measures model for the analysis of startle amplitudes and explicit measures (valence and arousal ratings). To correct for violations of sphericity across all calculations, the Greenhouse–Geisser procedure was utilised. Corrected values were reported for *p*-values in instances where sphericity was not met. Simple contrasts with Bonferroni corrections were used to determine the direction of any significant main effects.

## 3. Results

### 3.1. Self-Reported Aggression, Impulsivity and Self-Monitoring

Descriptive statistics of participant responses to the questionnaire can be seen in Table 2. Participant groups were divided based on a median split of BDHI total score. Individuals whose total score on the BDHI was above 29 were put into the ‘high’ group and those who scored below 29 were put into the ‘low’ group. There were no scores of 29 on the BDHI in our cohort. Mean ages did not significantly differ between groups. Importantly, one-way independent ANOVA showed that there was no significant difference between low and high groups with regards to Snyder total score (F(1,50) = 2.421, *p* = 0.126). However, analysis did reveal that those in the high aggressive group scored significantly higher in the impulsivity scale (F(1, 50) = 7.644, *p* = 0.008).

### 3.2. Explicit Responses

**Figure 1:**
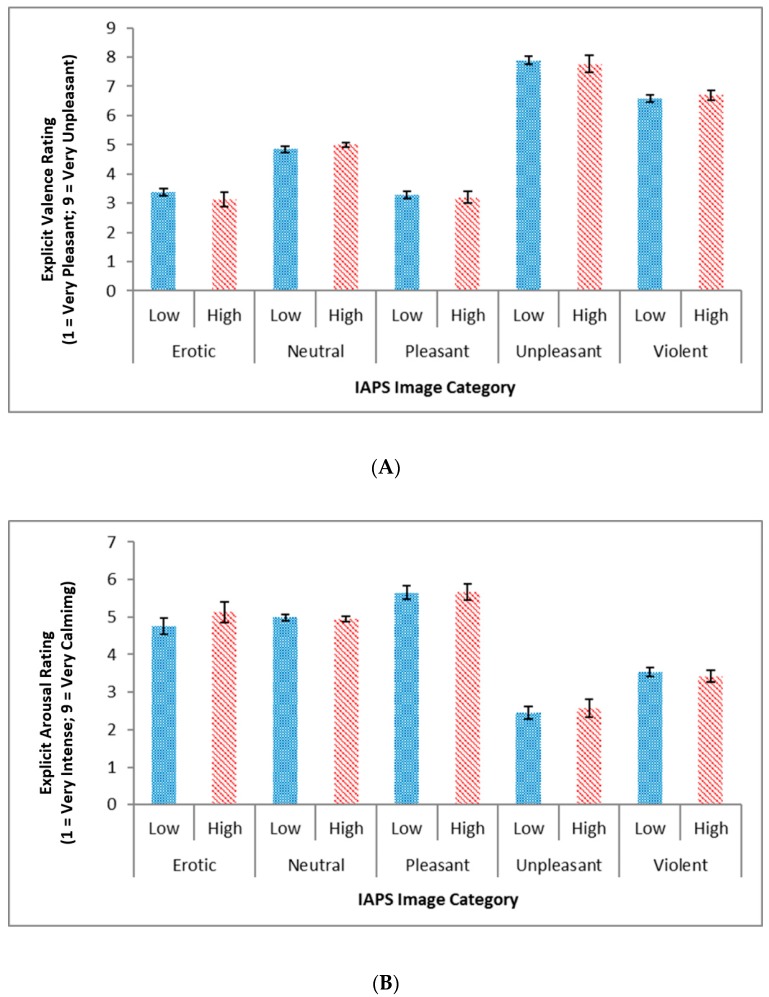
(**A**) Explicit valence rating and (**B**) explicit arousal rating for each emotion category across low and high aggression groups. Note no significant differences were noted in explicit valence and arousal ratings between both high and low aggression groups. IAPS: International Affective Picture System.

Results obtained from the general linear model for the explicit valence and arousal ratings did not show a significant group by emotion interaction for either valence scores (F(1.574) = 0.441, *p* = 0.597) or arousal scores (F(2.041) = 0.632, *p* = 0.536; see Figure 1A,B).

### 3.3. Physiological Measures

#### 3.3.1. Startle Reflex Modulation (SRM)

**Figure 2:**
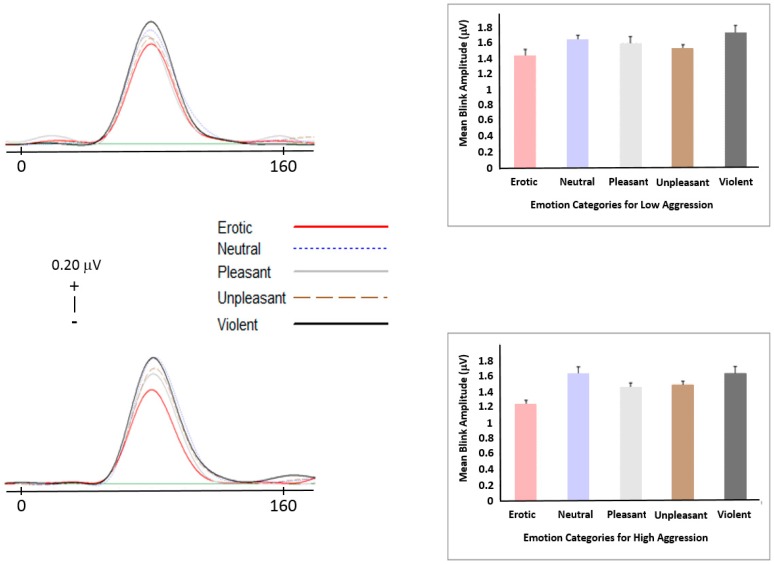
Startle reflex curves and column graphs for low (top graph) and high (bottom graph) aggression groups.

Results were only obtained for *N* = 50 participants due to faulty channels. Both excluded cases were from the low aggression group. Startle reflex modulation results showed no significant group effect of amplitude over any emotion condition (F(3.456) = 0.678, *p* = 0.587, *η*^2^ = 0.014, observed power = 0.203; see Figure 2). However, as can be seen in Figure 2, there are clear differences between the erotic and the violent conditions with all other conditions in between, a pattern that is expected based on prior SRM findings.

#### 3.3.2. Event-Related Potentials (ERPs)

**Figure 3:**
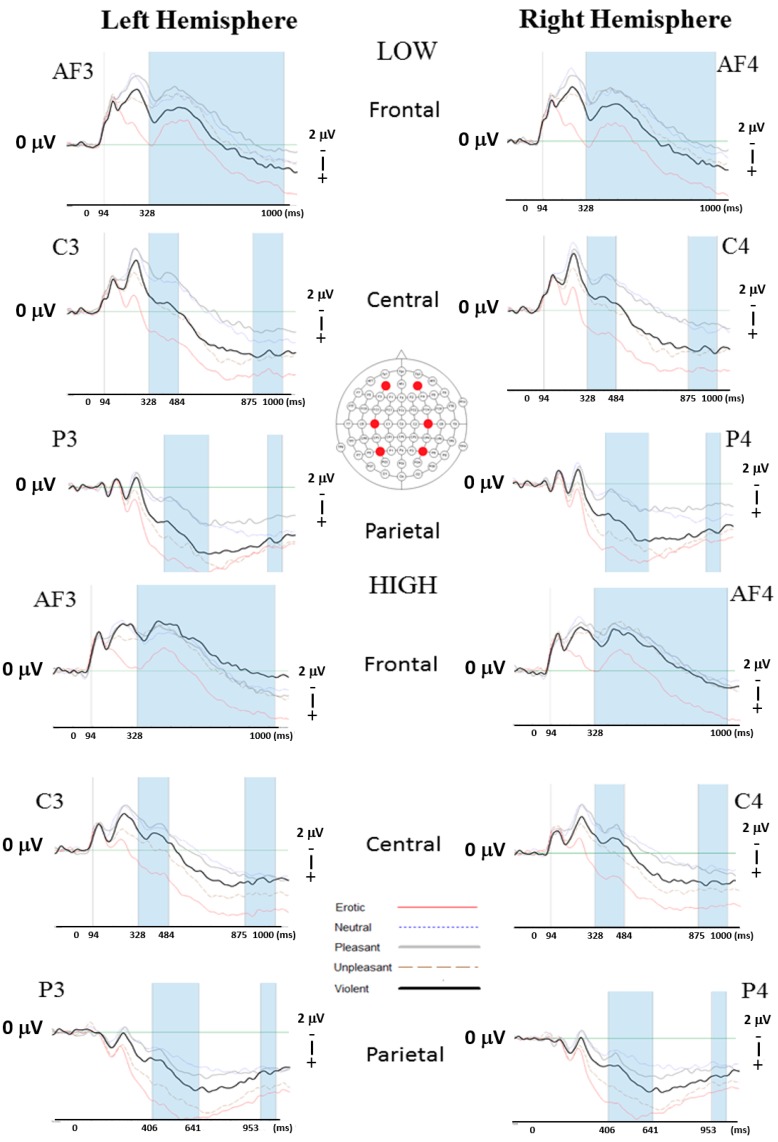
Event-related potential (ERP) effects representing frontal (AF3/AF4), central (C3/C4) and parietal (P3/P4) locations across all emotion categories for low and high aggression use groups. Note significant effects for ‘pleasant’ versus ‘violent’ emotion categories between 94 and 1000 ms in frontal areas of the brain and between 328 and 1000 ms in the central and parietal regions.

Following artefact correction, the number of trials included in the analysis for the ‘pleasant’ category ranged from 15–30 trials with a mean of 25.7. For the ‘violent’ category, the number of trials following artefact correction ranged from 14–30 trials with a mean of 26.10. Group by emotion effects reaching the threshold of significance were seen at 94 ms at frontal sites (F(3.76, 188.16) = 2.47, *p* = 0.05; *η*^2^ = 0.047; observed power = 0.678). No further significant within subject effects were noted in the EEG analysis across analysed electrode sites. Simple contrasts, however, revealed significant ERP group by emotion interaction effects for ‘pleasant’ versus ‘violent’ emotion categories between 484−1000 ms at frontal electrode sites. Contrasts further revealed significant effects between the same two emotion categories in central sites at 484 ms and 875–1000 ms and posterior sites at 406–1000 ms (Table 3; Figure 3). Significant effects across all time periods are shown; however, for the purposes of the current manuscript, only significant later ERP effects will be discussed. The absence of main group effects is interpreted as a result of rather focused ERP differences.

## 4. Discussion

The present study aimed to determine whether a particular sub-section of the general population, characterised only by their self-reported aggressive tendencies, differed in their conscious evaluation of emotional stimuli compared to objectively measured neurophysiological reaction to the same stimuli. By utilising a triangulation approach incorporating explicit rating measures (which require conscious, evaluative reasoning and responding via cortical brain processing), SRM (which is a measure of raw, affective information processing and is sensitive to sub-cortical brain processes), and EEG (which measures cortical graded potential changes of pyramidal cells), we were able to demonstrate that our participants differed in their subjective rating and their objectively measured neural responses to affective stimuli.

Event-related potential analysis showed significant group effects related to differences between the ‘pleasant’ and ‘violent’ emotion conditions across both hemispheres between both groups. Upon visual inspection of the curves in the frontal areas of the brain, the ERPs of the ‘pleasant’ and ‘violent’ emotion categories appear to follow a similar trajectory in the high aggression group which then separates in the left hemisphere approximately 800 ms post-stimulus onset. In the low aggression group, both curves (‘pleasant’ and ‘violent’) appear to follow a similar trajectory across both hemispheres but remain separate throughout the 1000 ms epoch studied. Much like the results obtained in the present study, Gagnon et al. [36] showed a similar response among aggressive individuals who responded with a larger N400 than their non-aggressive controls when both groups were presented with a critical word which violated a hostile expectation. The increased negative attenuation of the ERP amplitudes, noted in later time periods of the high aggression group to images portraying violence, may be due to increased attention payed to the image. Since individuals who are low aggressors are perhaps less likely to view violent images, the larger amplitude of their ERP is perhaps indicative of an increased motivational relevance due to the perception of threatening stimuli. Though not statistically significant, visual inspection of the curves obtained also showed that the high aggression group showed a decreased frontal response to the ‘violent’ images. These results can be compared with a study performed by Lu et al. [42] who demonstrated that highly aggressive individuals in their study reacted to threat-based stimuli (fearful faces) with a decreased fronto-central response compared with individuals with low aggression. The authors stated that this immediate reaction to threat-based stimuli was due to a fearful face representing a threat-based stimulus which requires a quick response. Heading more posteriorly to central and parietal sites, similar trends between the ‘pleasant’ and ‘violent’ emotion conditions can be seen. The ERP of the ‘violent’ emotion category appears to be more attenuated in the high aggressive group and so follows a similar trajectory to the ERP curve of the ‘pleasant’ condition, whereas the ERP curves of the ‘violent’ and ‘pleasant’ conditions remain separate in the low aggressive group. Although interesting, these findings are difficult to compare with previous studies on aggression. The vast majority of studies previously performed focused largely on the P300 component. They overwhelmingly show that highly aggressive individuals tend to show a more inhibited (smaller amplitude) P300 response than low aggressive individuals [31,50,51,52,53]. These studies posit the idea that the smaller P300 component may be due to cognitive deficits [51] or be associated with increases of aggression. Wang et al. [52] stated that this effect may imply a reduction in brain activity which has been known to reflect activation of the averse motivational system which can be linked to aggressive behaviour [54]. Present findings found statistically meaningful differences at the P300 component. Furthermore, rather prolonged statistically meaningful results were found post 300 ms. An explanation of the observed findings may be that, changes were seen in later time periods due to variations in meaningfulness of the displayed emotion images between the groups. Alternatively, as a consequence of life experience, high aggressors may be somewhat less responsive to violent stimuli, due to desensitisation effects. This may prevent them in engaging cognitively with the stimulus, resulting in the observed attenuated ERP amplitudes.

It is pertinent to note that studies performed by Stanford et al. and by Barratt et al. [33,34] showed that the reduced P300 component was only seen in impulsive aggressive individuals, prompting the idea that reduced P300 may only be characteristic of aggressive individuals who are impulsive and not premeditative [27]. The current study did not divide its sub-groups based on a combination of aggression and impulsivity scores. However, the authors did administer the BIS-11 [16] and found that participants in the high aggression group showed a higher mean score on the BIS-11 which may account for the diminished P300 in the ERPs for this group. Additionally, a study by Surguy and Bond [52], whilst also utilising the BDHI among a non-clinical population, revealed the same smaller amplitude P300 among higher aggressive individuals. They proposed that they may have seen greater differences in P300 amplitude had they recruited a cohort who may have scored more extreme on the scale. The mean BDHI scores within our high aggression cohort scored similarly, albeit slightly lower than those reported in the aforementioned study. Our study participants were younger than those studied by Surguy and Bond and were members of the general community, and not selected based on their aggression or hostility. It is therefore possible that our results may have been slightly diminished due to moderate rather than extreme levels of aggression in our sample as was also seen by Surguy and Bond [52]. 

As was expected, explicit valence and arousal ratings for all emotion categories showed no statistically significant differences between the low and high aggression groups. Several other studies have pinpointed the existence of similar results whereby conscious self-report often does not yield differing results among different sub-groups [24,38,54]. A possible explanation may be that the initial variation between high and low aggressive groups may not have been large enough to create a significant difference of conscious appraisal of emotional stimuli, as our cohort was drawn from a non-clinical population. Another possibility may be due to social desirability bias (SDB), whereby due to self-presentation concerns, respondents may have deliberately altered their ratings to more socially desirable ranges. The Snyder Self-Monitoring Scale was utilised to determine if either group was acutely more aware of how much they engaged in the expressive control of how they presented themselves in public. As both groups scored equally with no significant mean difference, it was evident that self-monitoring probably did not account for the non-significant results obtained from the explicit ratings. The conscious action-oriented nature of the valence and arousal ratings meant that our participants were required to consciously evaluate the emotion-laden images and determine their affective evaluation of the images presented. Knowingly, a multitude of implicit brain processes take place prior to initiating the conscious component of the task (explicit rating). This being so, it is evident that the two groups studied did not differ in their conscious interpretation, but they did differ in their objectively measured underlying brain processes elicited by the emotion images.

Startle reflex modulation also failed to show any significant main effects of group in the analysis. Perhaps self-reported aggression did not extend down to the sub-cortical areas of raw affective processing, accounting for the lack of significant differences between the high and low aggression groups. However, our study compared aggression using a median split within a non-clinical and non-forensic population. Therefore, the variation between high and low aggression within this cohort may have been insufficient to reveal differences in raw affective emotion processing, which SRM has demonstrated in comparisons between clinical and non-clinical populations [38,40,41,42,44]. SRM amplitude and latency have been shown to be higher and earlier respectively with increasing threat-related stimuli presentation with aggressive individuals generally presenting with an inhibited response to threat-related stimuli compared to matched controls [38]. Upon visual inspection of the startle curves, it can be seen that the ‘erotic’ and the ‘violent’ images have the smallest and largest peak amplitudes respectively across both groups. This finding is consistent with previous studies utilising this technique.

Despite the comprehensive nature of this study, there were unavoidable limitations. It should be noted that, by its nature, physiological measures may be a more sensitive measure of the physiological components of aggression than traditional self-report responses which are more sensitive to observable behaviours. This increased sensitivity does bring a potential statistical significance due to its inherent ability to pick up subtle changes in an individual’s physiology. Self-report data, in comparison, lacks the ability to determine such subtleties which, in an investigation such as this, puts any data sourced from it at a disadvantage. Furthermore, an emerging framework spearheaded by Thomas and Sharp, propose utilising a “mechanistic approach” which aims to integrate biological and psychological phenomena to determine how psychological functions are characterised by biological structures. This may have provided further insight into the biological underpinnings of the conscious behavioural responses detected and hence, provided us with a more meaningful comparison of recording techniques. The present study, however, was unfortunately designed whereby both measures were recorded in isolation from each other temporally, which would make this comparison inconsequential. Future iterations of similar studies may look to design their studies with multiple recording instruments at temporally meaningful time points to allow for a more mechanistic approach to determine the biological structural underpinnings of psychological functions.

Additionally, many studies highlight the differences between premeditated and impulsive aggressive individuals, with physiological biomarkers such as the P300 being indicative of impulsive and not premeditative aggressors. Therefore, it may have been preferable to group this cohort into ‘low BDHI’ and ‘low BIS11′ in order to determine physiological responses to impulsive aggressive individuals. As not all of the individuals who scored highly on the BDHI also scored highly on the BIS-11, this method would have meant we would have had to exclude several individuals from analysis and so, the authors chose to sacrifice this story for increased power during analysis and to look at aggression as a whole/unitary construct. Additionally, we utilised a modified version of the BDHI whereby we attempted to make it more apparent to our cohort that we were attempting to determine the extent of their aggressive traits with the hope they may intrinsically increase their self-monitoring. Future studies may wish to administer this questionnaire in its intended format as changing the format may have had an effect on self-monitoring as our cohort scored lower on the scale compared with similar studies which also utilised a normal population [53]. This also allows easier comparisons with previous literature providing more conclusive interpretations of results. Additionally, it may have been interesting to administer a questionnaire on social desirability (SD). As with aggression and violence, we are determining somewhat controversial and socially detested behaviours, and this is where social desirability has been shown to take precedence [7]. We aimed to decipher this by utilising the Snyder Self-Monitoring Scale, but as shown, the use of SD scales in questionnaire research influences results [55]. Future studies in this field may endeavour to utilise this construct. Authors should discuss the results and how they can be interpreted in perspective of previous studies and working hypotheses. The findings and their implications should be discussed in the broadest context possible. Future research directions may also be highlighted.

## 5. Conclusions

Taken together, our results indicate that it is possible to differentiate individuals within a normal population based on trait aggression by presenting them with affectively relevant stimuli. It may be evident that, as Surguy and Bond [52] postulate, the differentiation between this normal population may be more complex than what could be revealed when comparing highly aggressive individuals or patient populations to controls. Since we were utilising effects determined from exposure to affect-inducing images, it may have been interesting to determine how much violent imagery our cohort is generally exposed to (by way of violent video games, movies, television shows, etc.) in addition to their self-report aggression. We may have been able to determine whether the physiological findings were not only caused by conscious appraisal of aggressive tendencies but also if they were characteristic of desensitisation effects to violent stimuli. It is known that there are observable physiological desensitisation effects attributable to increased exposure to violent stimuli [50,56]. Due to speculation in the current study as to the relevance of self-report measures in determining aggression and aggressive tendencies, we believe that a multilevel approach involving measures which are sensitive to various levels of information processing is what is required in order to determine a more comprehensive model of understanding aggressive qualities in individuals. Future studies may look to further elaborate on the current study’s results and introduce exposure levels to violent stimuli as another variable when looking at conscious versus non-conscious emotional appraisal in this sub-group. The ultimate aim is to develop better models and markers to determine etiological variants to better inform clinical and research practice.

## Figures and Tables

**Table 1:** Demographic characteristics of sample.

Variable	*N*	(%)
**Marital Status**		
Married	1	1.9
Never married	39	75
DeFacto/living with a partner	12	23.1
**Highest Level of Completed Education**		
Secondary school completed	22	42.3
Trade qualification	1	1.9
University or another tertiary study	29	55.8
**Employment**		
Part time	7	13.5
Student	45	86.5
**Country of Birth**		
Australia	47	90.4
Other	5	9.6
Ethnicity *		
African	1	1.9
Asian	2	3.8
Caucasian	47	90.4
Other	1	1.9

* Not all sum due to non-responders.

**Table 2:** Descriptive statistics of total scores for low and high scoring groups based on the Buss–Durkee Hostility Inventory (BDHI) and in the total sample.

	Low BDHI Scorers (*N* = 26)		High BDHI Scorers (*N* = 26)		Total Sample (*N* = 52)	
	Mean	SD	Mean	SD	Mean	SD
Age	21.19	3.48	21.38	2.68	21.00	3.1
BDHI total	20.77	5.98	38.81	8.94	29.79	11.81
Snyder total	10.08	3.57	11.58	3.38	10.82	3.52
BIS-11 total	62.35 *	8.12	70.12 *	11.81	66.23	10.77

* Denotes significant differences between low and high aggression groups. SD: standard deviation; Snyder: Snyder Self-Monitoring Scale-Revised; BIS-11: Barratt Impulsivity Scale.

**Table 3:** Summary of significant ERP results for group by emotion across all significant time periods for pleasant versus violent emotion categories.

Electrode Sites	Time (ms)	*p*-Value
	94	0.05
	484	0.045
	641	0.011
**AF3/AF4**	719	0.008
	797	0.007
	875	0.014
	953	0.017
	1031	0.012
	484	0.026
**C3/C4**	875	0.036
	953	0.04
	1031	0.017
	406	0.044
	484	0.015
**P3/P4**	563	0.017
	641	0.034
	953	0.049
	1031	0.015

## References

[1] Siegel A., Victoroff J. (2009). Understanding human aggression: New insights from neuroscience. Int. J. Law Psychiatry.

[2] Barratt E.S., Monahan J., Steadman H. (1994). Impulsiveness and aggression. Violence Ment. Disord. Dev. Risk Assess..

[3] Eron L.D. (1987). The development of aggressive behavior from the perspective of a developing behaviorism. Am. Psychol..

[4] American Psychiatric Association (2013). Diagnostic and Statistical Manual of Mental Disorders (DSM-5).

[5] (1993). The ICD-10 Classification of Mental and Behavioural Disorders: Diagnostic Criteria for Research.

[6] Fulmer S.M., Frijters J.C. (2009). A Review of Self-Report and Alternative Approaches in the Measurement of Student Motivation. Educ. Psychol. Rev..

[7] Van de Mortel T.F. (2008). Faking it: Social desirability response bias in self-report research. Aust. J. Adv. Nurs..

[8] Hebert J.R., Clemow L., Pbert L., Ockene I.S. (1995). Social Desirability Bias in Dietary Self-Report May Compromise the Validity of Dietary Intake Measures. Int. J. Epidemiol..

[9] Kelly C.A., Soler-Hampejsek E., Mensch B.S., Hewett P.C. (2013). Social desirability bias in sexual behavior reporting: Evidence from an interview mode experiment in rural Malawi. Int. Perspect. Sex. Reprod. Health.

[10] Walla P. (2011). Non-Conscious Brain Processes Revealed by Magnetoencephalography (MEG). Magnetoencephalography.

[11] Walla P., Brenner G., Koller M. (2011). Objective Measures of Emotion Related to Brand Attitude: A New Way to Quantify Emotion-Related Aspects Relevant to Marketing. PLoS ONE.

[12] Berridge K., Winkielman P. (2003). What is an unconscious emotion? (The case for unconscious “liking”). Cogn. Emot..

[13] Winkielman P., Berridge K.C. (2004). Unconscious Emotion. Curr. Dir. Psychol. Sci..

[14] Tamietto M., De Gelder B. (2010). Neural bases of the non-conscious perception of emotional signals. Nat. Rev. Neurosci..

[15] Moyer K.E. (1968). Kinds of aggression and their physiological basis. Commun. Behav. Biol..

[16] Patton J.H., Stanford M.S., Barratt E.S. (1995). Factor structure of the barratt impulsiveness scale. J. Clin. Psychol..

[17] Walla P., Koller M. (2015). Emotion is not What You Think It Is: Startle Reflex Modulation (SRM) as a Measure of Affective Processing in NeuroIs. Information Systems and Neuroscience.

[18] Lang P.J., Bradley M.M., Cuthbert B.N. (1990). Emotion, attention, and the startle reflex. Psychol. Rev..

[19] Mavratzakis A., Herbert C., Walla P. (2016). Emotional facial expressions evoke faster orienting responses, but weaker emotional responses at neural and behavioural levels compared to scenes: A simultaneous EEG and facial EMG study. NeuroImage.

[20] Geiser M., Walla P. (2011). Objective Measures of Emotion During Virtual Walks through Urban Environments. Appl. Sci..

[21] Koller M., Walla P. (2015). Towards Alternative Ways to Measure Attitudes Related to Consumption: Introducing Startle Reflex Modulation. J. Agric. Food Ind. Organ..

[22] Nesbitt K., Blackmore K., Hookham G., Kay-Lambkin F., Walla P. (2015). Using the Startle Eye-Blink to Measure Affect in Players. Serious Games Analytics.

[23] Walla P., Koller M., Meier J.L. (2014). Consumer neuroscience to inform consumers-physiological methods to identify attitude formation related to over-consumption and environmental damage. Front. Hum. Neurosci..

[24] Grahl A., Greiner U., Walla P. (2012). Bottle Shape Elicits Gender-Specific Emotion: A Startle Reflex Modulation Study. Psychology.

[25] Allen N.B., Trinder J., Brennan C. (1999). Affective startle modulation in clinical depression: Preliminary findings. Biol. Psychiatry.

[26] Walla P., Koller M., Brenner G., Bosshard S. (2017). Evaluative conditioning of established brands: Implicit measures reveal other effects than explicit measures. J. Neurosci. Psychol. Econ..

[27] Patrick C.J. (2008). Psychophysiological correlates of aggression and violence: An integrative review. Philos. Trans. R. Soc. B: Biol. Sci..

[28] Donchin E., Coles M.G.H. (1988). Is the P300 component a manifestation of context updating?. Behav. Brain Sci..

[29] Hillyard S.A., Kutas M. (1983). Electrophysiology of Cognitive Processing. Annu. Rev. Psychol..

[30] Harmon-Jones E., Barratt E.S., Wigg C. (1997). Impulsiveness, aggression, reading, and the P300 of the event-related potential. Pers. Individ. Differ..

[31] Bond A.J., Surguy S.M. (2000). Relationship between attitudinal hostility and P300 latencies. Prog. Neuro-Psychopharmacol. Biol. Psychiatry.

[32] Gerstle J.E., Mathias C.W., Stanford M.S. (1998). Auditory P300 and self-reported impulsive aggression. Prog. Neuro-Psychopharmacol. Biol. Psychiatry.

[33] Stanford M.S., Houston R.J., Villemarette-Pittman N.R., Greve K.W. (2003). Premeditated aggression: Clinical assessment and cognitive psychophysiology. Pers. Individ. Differ..

[34] Barratt E.S., Stanford M.S., Kent T.A., Alan F. (1997). Neuropsychological and cognitive psychophysiological substrates of impulsive aggression. Biol. Psychiatry.

[35] Koller M., Walla P. Measuring Affective Information Processing in Information Systems and Consumer Research—Introducing Startle Reflex Modulation. Proceedings of the 23th International Conference on Information Systems (ICIS).

[36] Gagnon J., Aubin M., Emond F.C., Derguy S., Brochu A.F., Bessette M., Jolicoeur P. (2016). An ERP study on hostile attribution bias in aggressive and nonaggressive individuals. Aggress. Behav..

[37] Schupp H.T., Flaisch T., Stockburger J., Junghöfer M. (2006). Emotion and attention: Event-related brain potential studies. Motivation—Theory, Neurobiology and Applications.

[38] Patrick C.J., Bradley M.M., Lang P.J. (1993). Emotion in the criminal psychopath: Startle reflex modulation. J. Abnorm. Psychol..

[39] Vaidyanathan U., Hall J.R., Patrick C.J., Bernat E.M. (2011). Clarifying the role of defensive reactivity deficits in psychopathy and antisocial personality using startle reflex methodology. J. Abnorm. Psychol..

[40] Dawson M.E., Hazlett E.A., Filion D.L., Nuechterlein K.H., Al E. (1993). Attention and schizophrenia: Impaired modulation of the startle reflex. J. Abnorm. Psychol..

[41] Kumari V., Das M., Hodgins S., Zachariah E., Barkataki I., Howlett M., Sharma T. (2005). Association between violent behaviour and impaired prepulse inhibition of the startle response in antisocial personality disorder and schizophrenia. Behav. Brain Res..

[42] Hazlett E.A., Speiser L.J., Goodman M., Roy M., Carrizal M., Wynn J.K., Williams W.C., Romero M., Minzenberg M.J., Siever L.J. (2007). Exaggerated Affect-Modulated Startle During Unpleasant Stimuli in Borderline Personality Disorder. Biol. Psychiatry.

[43] Walla P., Rosser L., Scharfenberger J., Duregger C., Bosshard S. (2013). Emotion Ownership: Different Effects on Explicit Ratings and Implicit Responses. Psychology.

[44] Buss A.H., Durkee A. (1957). An inventory for assessing different kinds of hostility. J. Consult. Psychol..

[45] LimeSurvey Partner Services www.limesurvey.com.

[46] Snyder M., Gangestad S. (1986). On the nature of self-monitoring: Matters of assessment, matters of validity. J. Pers. Soc. Psychol..

[47] Lang P., Bradley M., Cuthbert B. (2008). International Affective Picture System (IAPS): Affective Ratings of Pictures and Instruction Manual.

[48] Bartholow B.D., Bushman B.J., Sestir M.A. (2006). Chronic violent video game exposure and desensitization to violence: Behavioral and event-related brain potential data. J. Exp. Soc. Psychol..

[49] Kunaharan S., Halpin S., Sitharthan T., Bosshard S., Walla P. (2017). Conscious and Non-Conscious Measures of Emotion: Do They Vary with Frequency of Pornography Use?. Appl. Sci..

[50] Zheng Y., Li J.H. (2014). Attentional Bias in Individuals with Different Level of Implicit/Explicit Aggression: Behavioral and ERP Evidence. J. Psychol. Sci..

[51] Wang Y., Zhao Y., Qiu J., Ybarra O., Liu L., Huang Y. (2012). Neural Correlates of Aggression among Individuals from Low and High Socioeconomic Status: An ERP Study. Int. J. Psychol. Stud..

[52] Surguy S.M., Bond A.J. (2006). P300 to emotionally relevant stimuli as an indicator of aggression levels. Aggress. Behav..

[53] Meffert H., Gazzola V., Boer J.A.D., Bartels A.A.J., Keysers C. (2013). Reduced spontaneous but relatively normal deliberate vicarious representations in psychopathy. Brain.

[54] Krumpal I. (2013). Determinants of social desirability bias in sensitive surveys: A literature review. Qual. Quant..

[55] Bailey K., West R., Anderson C.A. (2011). The association between chronic exposure to video game violence and affective picture processing: An ERP study. Cogn. Affect. Behav. Neurosci..

[56] Lu H., Wang Y., Xu S., Wang Y., Zhang R., Li T. (2015). Aggression differentially modulates brain responses to fearful and angry faces: An exploratory study. Neuroreport.

